# Plasma Exosome-Enriched Extracellular Vesicles From Lactating Mothers With Type 1 Diabetes Contain Aberrant Levels of miRNAs During the Postpartum Period

**DOI:** 10.3389/fimmu.2021.744509

**Published:** 2021-10-08

**Authors:** Caroline Frørup, Aashiq H. Mirza, Reza Yarani, Lotte B. Nielsen, Elisabeth R. Mathiesen, Peter Damm, Jens Svare, Christian Engelbrekt, Joachim Størling, Jesper Johannesen, Henrik B. Mortensen, Flemming Pociot, Simranjeet Kaur

**Affiliations:** ^1^ Translational Type 1 Diabetes Research, Steno Diabetes Center Copenhagen, Gentofte, Denmark; ^2^ Department of Clinical Medicine, Faculty of Health and Medical Sciences, University of Copenhagen, Copenhagen, Denmark; ^3^ Department of Pharmacology, Weill Cornell Medical, New York, NY, United States; ^4^ Copenhagen Diabetes Research Center (CPH-DIRECT), Department of Pediatrics, Herlev and Gentofte Hospital, Herlev, Denmark; ^5^ Department of Endocrinology, Rigshospitalet, Copenhagen, Denmark; ^6^ Center for Pregnant Women with Diabetes, Rigshospitalet, Copenhagen, Denmark; ^7^ Department of Obstetrics, Rigshospitalet, Copenhagen, Denmark; ^8^ Department of Obstetrics, Herlev and Gentofte Hospital, Herlev, Denmark; ^9^ Department of Chemistry, Technical University of Denmark, Lyngby, Denmark; ^10^ Department of Biomedical Sciences, Faculty of Health and Medical Sciences, University of Copenhagen, Copenhagen, Denmark

**Keywords:** extracellular vesicles, exosomes, miRNAs, plasma, small RNA-Seq, type 1 diabetes

## Abstract

Type 1 diabetes is an immune-driven disease, where the insulin-producing beta cells from the pancreatic islets of Langerhans becomes target of immune-mediated destruction. Several studies have highlighted the implication of circulating and exosomal microRNAs (miRNAs) in type 1 diabetes, underlining its biomarker value and novel therapeutic potential. Recently, we discovered that exosome-enriched extracellular vesicles carry altered levels of both known and novel miRNAs in breast milk from lactating mothers with type 1 diabetes. In this study, we aimed to characterize exosomal miRNAs in the circulation of lactating mothers with and without type 1 diabetes, hypothesizing that differences in type 1 diabetes risk in offspring from these groups are reflected in the circulating miRNA profile. We performed small RNA sequencing on exosome-enriched extracellular vesicles extracted from plasma of 52 lactating mothers around 5 weeks postpartum (26 with type 1 diabetes and 26 age-matched controls), and found a total of 2,289 miRNAs in vesicles from type 1 diabetes and control libraries. Of these, 176 were differentially expressed in plasma from mothers with type 1 diabetes (167 upregulated; 9 downregulated, using a cut-off of abs(log2FC) >1 and FDR adjusted p-value <0.05). Extracellular vesicles were verified by nanoparticle tracking analysis, transmission electron microscopy and immunoblotting. Five candidate miRNAs were selected based on their involvement in diabetes and immune modulation/beta-cell functions: hsa-miR-127-3p, hsa-miR-146a-5p, hsa-miR-26a-5p, hsa-miR-24-3p and hsa-miR-30d-5p. Real-time qPCR validation confirmed that hsa-miR-146a-5p, hsa-miR-26a-5p, hsa-miR-24-3p, and hsa-miR-30d-5p were significantly upregulated in lactating mothers with type 1 diabetes as compared to lactating healthy mothers. To determine possible target genes and affected pathways of the 5 miRNA candidates, computational network-based analyses were carried out with TargetScan, mirTarBase, QIAGEN Ingenuity Pathway Analysis and PantherDB database. The candidates showed significant association with inflammatory response and cytokine and chemokine mediated signaling pathways. With this study, we detect aberrant levels of miRNAs within plasma extracellular vesicles from lactating mothers with type 1 diabetes during the postpartum period, including miRNAs with associations to disease pathogenesis and inflammatory responses.

## Introduction

Type 1 diabetes is an immune-mediated disease, characterized by immune-cell targeting of the insulin producing beta cells of the pancreatic islets of Langerhans, leading to their demise and resulting insulin deficiency (1, 2). Several studies have emphasized the occurrence of residual beta-cell mass, years after diagnosis, suggesting a potential for beta-cell preservation and regeneration in type 1 diabetes (3–5). This calls for further exploration into understanding the molecular drivers in type 1 diabetes. And while this to date remains largely unknown, it has been well established that some environmental and genetic risk factors can both contribute to and protect against disease development (2, 6).

Remarkably, the risk of developing type 1 diabetes in offspring of parents with preexisting type 1 diabetes seems to be different dependent on which parent is affected, i.e. the frequency is lower if the mother has type 1 diabetes, compared to the father (7), indicating an alteration in the genetic or molecular milieu in women with type 1 diabetes during pregnancy and postpartum. This could associate to the major molecular changes occurring during and after pregnancy, e.g. pro-inflammatory cytokines, c-peptide levels, pregnancy-associated growth hormones and lactating hormones, affecting both the woman and the developing fetus or breastfed infant (8, 9). Some changes are normalized at delivery or shortly thereafter, while others purposely persist (8). Interestingly, breastfeeding has shown to be a protective factor against the infant’s risk of developing type 1 diabetes (10–12). However, the molecular mechanisms underlying this remain largely unexplored, and has never been studied in a setting comparing healthy women and women with preexisting type 1 diabetes. We speculate that circulating factors hold the ability to modulate type 1 diabetes risk in offspring of mothers with type 1 diabetes, still reflected after delivery.

Recently, we investigated the breast milk from lactating mothers with and without with type 1 diabetes, focusing on the exosome-enriched extracellular vesicle microRNA (miRNA) signature (13). We found that the breast milk from mothers with type 1 diabetes carry altered levels of both known and novel miRNAs, compared to healthy mothers, and that these miRNAs were associated with potentially immunomodulatory effects in the breastfed infant (13). Several other studies have previously highlighted the implication of distorted miRNA levels in the pathogenesis of type 1 diabetes (14–18). Particularly, the exosomal miRNAs are currently under investigation for their use as potential disease biomarkers (19, 20).

miRNAs target around 60% of all transcribed genes, exerting their function within the intracellular compartments of various cells (21, 22). However, they are also extensively being transported out of the cells in membrane-bound particles, broadly known as extracellular vesicles: the ~30-150 nm exosomes; the ~100-1,000 nm microvesicles; and the up to 5,000 nm large apoptotic bodies (19, 23, 24). While apoptotic bodies are thought to merely be a product of decayed cell packaging, microvesicles, arising from pinching off the cell membrane, and exosomes, formed by intracellular invagination, are both actively released by the cells. These are known to carry a broad variety of molecular cargo; protein, lipid and RNA-species, including unique miRNA profiles (19, 23). Therefore, these vesicles have been linked to cell-to-cell signaling, transporting specific and actively selected miRNAs from their parent cell to a recipient cell or tissue, while ensuring high abundance, availability and protection against degradation of the miRNAs (25, 26).

In this study, we aimed to characterize putative differences in circulating miRNA species in mothers with or without type 1 diabetes during the postpartum period. We hypothesized that differences in type 1 diabetes risk in offspring from these groups are reflected in the circulating miRNA profile. To investigate this, we extracted extracellular vesicles and its miRNA content from plasma samples of the study participants and performed small RNA sequencing (RNA-Seq) with unique molecular identifiers (UMIs). With this novel technology, we identify more than 2,000 miRNAs, 176 being differentially expressed in lactating mothers with type 1 diabetes.

## Materials and Methods

### Study Design and Sample Collection

**Figure 1:**
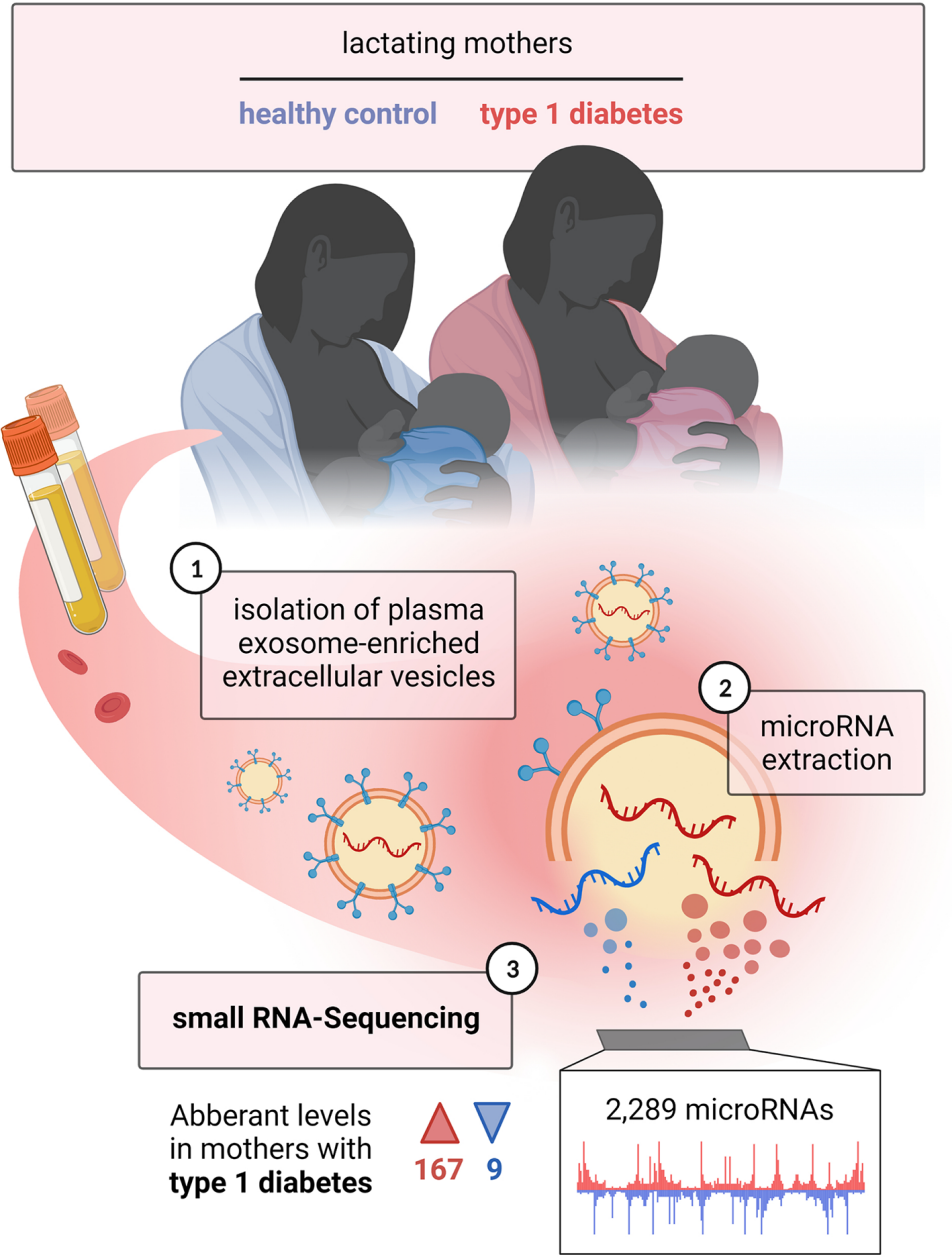
Study overview. Lactating mothers with and without type 1 diabetes were included in the study, from whom blood samples were taken; 1) isolation of plasma exosome-enriched extracellular vesicles was performed; 2) microRNAs were extracted; 3) small RNA sequencing was carried out. The figure is intended to briefly summarize the study design, workflow and results in a simplified schematic. Created with BioRender.com

The study was approved by the Ethical Committee for the Capital Region, Denmark (H-4-2013-008) and performed in accordance with the Declaration of Helsinki. All the participants signed the informed consent. The study included 52 lactating mothers; 26 mothers with pre-existing type 1 diabetes and 26 healthy mothers. Inclusion criteria were healthy, normal birth-weight infants born at gestational age ≥ 37 weeks and continuing breastfeeding. Exclusion criteria were type 2 diabetes, smoking, and complications during delivery. All mothers were recruited from Herlev and Gentofte Hospital and Rigshospitalet. None of the participants were treated for infections, inflammation or presented with symptoms thereof. None of the participants suffered from diabetes related complications. Clinical characteristics (age, BMI, blood glucose, HbA_1c_, insulin dose, gestational age at delivery) were collected for all participants [**Table 1** (13)]. The blood samples were collected around five weeks after delivery from a cubital vein into EDTA Vacutainer tubes and all variables were measured. The samples were immediately centrifuged at 2,500 x g at 4°C for 10 min. and plasma were collected and stored in aliquots at −80°C prior to experimental processing and analysis. The study design is outlined in (**Figure 1**).

**Table 1:** Clinical characteristics with mean ± SD and calculated p-values between the 52 participants; lactating mothers with type 1 diabetes (n=26); lactating healthy mothers (n=26).

Clinical characteristics			
Characteristic	Control (n=26)	type 1 diabetes (n=26)	p-value
Age (years)	31.8 ± 4.5	32.2 ± 4.8	0.74
BMI (kg/m^2^)	26.9 ± 4.5	27.0 ± 4.4	0.95
BG (mmol/l)	5.6 ± 0.7	10.0 ± 4.9	6.78E-05
HbA_1c_ (mmol/mol)	27.7 ± 4.1	42.4 ± 8.1	7.30E-08
Insulin dose (IU/24 hours)	–	31.7 ± 12.3	–
Sampling (days after delivery)	36.9 ± 8.5	35.8 ± 7.7	0.76
Gestational age at delivery (weeks)	39.8 ± 1.2	37.9 ± 0.6	1.84E-06

BMI, body mass index; BG, blood glucose.

### Extraction and Characterization of Plasma Exosome-Enriched Extracellular Vesicles

Briefly, 3 ml plasma per sample was diluted with 3 ml 1X PBS (pH 7.5) followed by centrifugation at 5,000 x g for 3 min. Next, 3.5 ml of the supernatant was filtered with a nitrocellulose filter (0.8 µm pore size) (VWR, Radnor, PA, USA). The extracellular vesicles were extracted by ExoEasy Serum Plasma Kit (Qiagen, Valencia, CA, USA) according to the manufacturer’s protocol.

Vesicle concentration per ml and size distribution were determined using Malvern NanoSight LM10 instrument (Malvern Panalytical technologies, Malvern, UK) and analyzed using nanoparticle tracking analysis (NTA) 3.1 Build 3.1.46. Extracellular vesicles were diluted in 1X PBS buffer for the analysis. Visualization of vesicles was carried out using transmission electron microscopy (TEM) by staining with 2% phosphotungstic acid and imaged with a CM 100 electron microscopy at 100 kV.

The presence of exosome particles was determined by immunoblotting of exosome markers CD63, CD9, CD81 and heat shock protein 70 (HSP70) and microvesicle markers Annexin A1 and ADP-ribosylation factor 6 (ARF6) in the plasma-derived extracellular vesicle samples from mothers with and without type 1 diabetes. Proteins were extracted after vesicle disintegration with cold RIPA Lysis and Extraction Buffer (Thermo Fisher Scientific, Waltham, MA, USA) supplemented with Halt protease and phosphatase inhibitor cocktail (Thermo Fisher Scientific). Protein concentrations were determined using the DC Protein Assay (Bio-Rad, Hercules, CA, USA) and 25 µg/well were denatured with 2 µl dithiothreitol solution (AppliChem, Germany) and/or 5 µl NuPage LDS buffer (Thermo Fisher Scientific), dependent on protein of interest, at 75°C for 10 min. Immunoblotting was carried out by electrophoresis with Bolt 4–12% Bis-Tris Plus gels (Thermo Fisher Scientific) in MES running buffer (Thermo Fisher Scientific). Proteins were transferred onto a 0.45 µm nitrocellulose membrane (Thermo Fisher Scientific) and blocked in 5% skim milk in Tris-buffered saline with 0.1% Tween 20. Membranes were probed with primary antibodies for exosome surface markers: anti-CD63 diluted 1:500, anti-CD9 diluted 1:500, anti-CD81 diluted 1:1000, anti-HSP70 diluted 1:3000, anti-Annexin A1 diluted 1:1000, anti-ARF6 diluted 1:500 (all from Invitrogen, Carlsbad, CA, USA) and following secondary probing with HRP-conjugated anti-rabbit IgG antibody (Cell Signaling, Danvers, MA, USA) diluted 1:2000 and anti-mouse IgG antibody diluted 1:1000 (Cell Signaling). Bands were visualized by chemiluminescence with LumiGLO and peroxide reagents (Cell Signaling) and a FUJI LAS4000 Imager (GE Healthcare Chicago, IL, USA).

### Small RNA Sequencing and Analysis

The exosomal RNA was extracted by exoRNeasy Serum Plasma Kit (Qiagen), and small RNA sequencing was performed by Qiagen’s QIAseq miRNA sequencing platform (NextSeq 500), generating only miRNA specific UMIs. Prior to the small RNA library preparation, the quality of RNA extracted from exosome-enriched extracellular vesicles was assessed by miScript II RT Kit (Qiagen) and miScript SYBR Green PCR Kit (Qiagen) following manufacturer’s instructions (**Supplementary Figure 1A**). In addition, RNA sample quality was assessed using miScript miRNA QC PCR Array (Qiagen) (**Supplementary Figure 1B**). Hemolysis was checked with the assessment of the relative expression of the erythrocyte specific hsa-miR-451a and plasma stable hsa-miR-23a-3p (27). Libraries were prepared using QIAseq miRNAseq library kit following the manufacturer’s instructions. The raw fastq files were analyzed using GeneGlobe Data Analysis Software, and the reads were processed as follows. First, the miRNA entries were calibrated based on identical or near-identical sequences in miRBase mature database. Reads were then processed by trimming off the 3’ adapter and low-quality bases using cutadapt (cutadapt.readthedocs.io/en/stable/guide.html). Following trimming, UMI sequences were identified. Reads with less than 16 bp insert sequences or less than 10 bp UMI sequences were discarded. Reads were aligned using bowtie (bowtiebio.sourceforge.net/index.shtml), with up to two mismatches allowed. Read counts for each RNA category (miRBase mature, miRBase hairpin, piRNA, tRNA, rRNA, mRNA and other RNA) were calculated from the mapping results using miRBase V21 and piRNABank. All reads assigned to a particular miRNA were counted, and the associated UMIs were aggregated to count unique molecules. The UMI reads were normalized using Trimmed Mean of M (TMM) method in edgeR (28). To filter the lowly expressed miRNAs, a cut-off of UMI >10 in at least 40% of the samples was used. The differential expression analysis was performed on the two groups using GLM approach in edgeR. The differentially expressed miRNAs were identified using a cut-off of abs(log2FC) >1 and FDR adjusted p-value <0.05. The sample size (n=26 in each group) had a power >85% to demonstrate a 0.5-fold change between groups with a significance level of 5%. All statistical analyses were conducted using various bioconductor packages in R (29).

### miRNA Expression Profiling by Real-Time qPCR in Plasma

To verify the RNA-Seq data, we wanted to select few miRNA candidates based on top hit differentially expression and known roles in immune-modulating pathways and beta-cell functions, for validation by real-time qPCR: hsa-miR-127-3p (477889_mir), hsa-miR-146a-5p (478399_mir), hsa-miR-26a-5p (477995_mir), hsa-miR-24-3p (477992_mir) and hsa-miR-30d-5p (478606_mir). Limited material available after exosome isolation allowed this to be carried out on 46 of the 52 samples (n=23 for each group). miRNAs were extracted and purified from the plasma of mothers with and without type 1 diabetes using the miRNeasy serum/plasma kit (Qiagen). RNA was quantified by NanoDrop Spectrophotometry (Thermo Fisher Scientific) and cDNA synthesis was performed using the TaqMan Advanced miRNA cDNA Synthesis Kit (Thermo Fisher Scientific). TaqMan Advanced miRNA Assays and TaqMan Advanced Master Mix (Thermo Fisher Scientific) were used to validate the 5 selected miRNAs by real-time qPCR on the thermal cycler CFX384 system (Bio-Rad), with conditions: 20 sec. at 95°C and 40 cycles of 1 sec. at 95°C and 20 sec. at 60°C. Data were analyzed using the 2^-ΔCt^ method (30) and normalized to a geomean of stable and highly expressed reference genes: hsa-miR-16-5p (477860_mir) and hsa-miR-30e-5p (479235_mir).

### Network and Pathway Analysis of miRNA Targets

The target genes for the 5 differentially expressed miRNAs that were selected for validation were retrieved using TargetScan (31) and mirTarBase (32). In total, 36,665 targets were retrieved for the 5 miRNAs: hsa-miR-127-3p, hsa-miR-146a-5p, hsa-miR-26a-5p, hsa-miR-24-3p and hsa-miR-30d-5p. Targets that were common between TargetScan and miRTarBase (n=431) were selected for further analysis. To identify potential molecular interactions, networks and relationships for these 5 differentially expressed miRNAs, we next performed the Core Analysis using QIAGEN Ingenuity Pathway Analysis (IPA) software (33). The network of differentially expressed miRNAs and their targets was visualized and grouped using the IPA module Path Designer. The PantherDB classification system (34) was used to determine pathway based functional annotations of the gene targets belonging to the 5 miRNAs. This was done using a binomial test, with FDR for multiple corrections.

### Statistical Analysis

All the statistical analyses and graphs were created using bioconductor packages in R and GraphPad Prism version 8. Correlations between the differentially expressed exosome-enriched extracellular vesicle-derived miRNAs from plasma (log2UMIs) and HbA_1c_ (mmol/mol) of mothers with type 1 diabetes were calculated using a two-tailed Pearson’s correlation and adjusted p-value <0.05 by correcting for multiple testing with Benjamini-Hochberg procedure. Real-time qPCR-based validation of selected miRNA candidates was evaluated using Student’s *t* test. P-values <0.05 were considered statistically significant. Validation data are expressed as means ± SEM.

## Results

### Study Population Characteristics

The clinical characteristics of the study population of the 52 mothers are presented in (**Table 1**). There was no difference in age or body weight between the two groups of mothers. Expectedly, blood glucose and HbA_1c_ were different between the groups. Also, gestational age at delivery was lower for women with type 1 diabetes. Quality control showed stable hsa-miR-451a and hsa-miR-23a-3p ratios, verifying that samples were not affected by hemolysis.

### Characterization of Exosome-Enriched Extracellular Vesicles Derived From Plasma

To investigate the particle population enriched in the plasma-derived extracellular vesicle samples, we performed NTA and TEM. Size and concentration of the extracellular vesicles were specified by NTA to cover vesicles, including adsorbates and electric double layer, with a mean hydrodynamic diameter of 240 nm and concentration of 3.7 x 10^9^ particles/ml (**Supplementary Figure 2A**). TEM verified the enrichment of particles smaller than 200 nm in diameter (**Supplementary Figure 2B**). The expression of exosome surface markers CD63, CD9, CD81 and HSP70 were verified in the plasma-derived extracellular vesicles by immunoblotting (**Supplementary Figure 2C**). Microvesicle surface markers Annexin A1 and ARF6 were not detectable in the plasma-derived extracellular vesicle samples (data not shown). This supports that the isolation predominantly comprises exosome particles. Antibodies were validated in supplementary samples to account for specificity bias. Strong non-specific binding was detected by the ARF6 antibody.

### miRNA Expression Profiles From Exosome-Enriched Extracellular Vesicles Derived From Plasma

**Figure 2:**
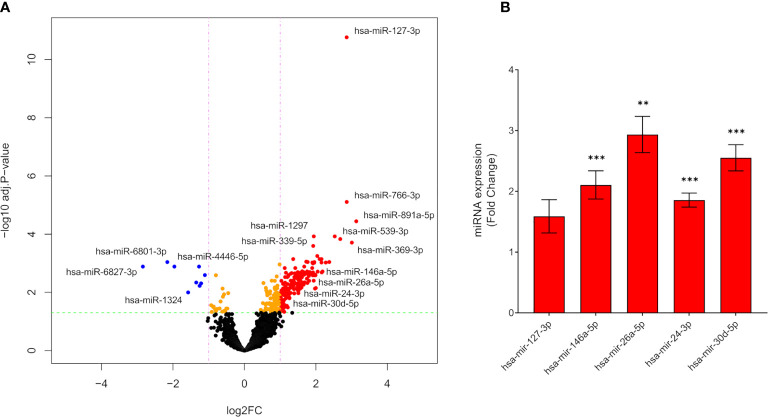
Differentially expressed miRNAs from lactating mothers with type 1 diabetes compared to lactating healthy mothers. **(A)** Volcano plot of differentially expressed miRNAs in mothers with type 1 diabetes compared to control mothers by RNA-Seq (upregulated miRNAs: red dots, downregulated miRNAs: blue dots). Dotted red lines represent the log2FC cut-off., dotted green lines represent -log10 adjusted p-value cut-off (n=52). **(B)** mRNA expression by real-time qPCR of miRNA hsa-miR-127-3p, hsa-miR-146a-5p, hsa-miR-26a-5p, hsa-miR-24-3p and hsa-miR-30d-5p in plasma from mothers with type 1 diabetes compared to control. The mRNA expression levels are presented as Fold Change +/- SEM based on 2^-ΔCt^ (n=46), **p < 0.01, ***p < 0.001.

From small RNA-Seq, a yield of miRNA-specific UMIs of 1.5 million were obtained. Library sizes, sample correlations and multidimensional scaling (MDS) plot are shown in **(Supplementary Figures 3A–C**). Quality control results are shown in (**Supplementary Figure 1**). A total of 2,289 miRNAs were detected in the libraries from the two groups using a cut-off of >10 UMI counts in at least 40% of the samples. In total, 176 differentially expressed miRNAs were identified when comparing lactating mothers with type 1 diabetes with lactating healthy mothers; 167 upregulated; 9 downregulated [**Figure 2A**, abs(log2FC) ≥1, FDR adjusted p-value <0.05].

### hsa-miR-146a-5p, -26a-5p, -24-3p and -30d-5p Are Upregulated in Plasma of Mothers With Type 1 Diabetes

We selected 5 significantly upregulated miRNAs based on disease relevance and a known role in beta cell and immune modulation for validation. By real-time qPCR, hsa-miR-127-3p, hsa-miR-146a-5p, hsa-miR-26a-5p, hsa-miR-24-3p and hsa-miR-30d-5p were validated in plasma. hsa-miR-146a-5p; 2.1-fold p<0.001, hsa-miR-26a-5p; 2.9-fold p<0.01, hsa-miR-24-3p; 1.9-fold p<0.001 and hsa-miR-30d-5p; 2.6-fold p<0.001, were significantly upregulated in plasma from mothers with type 1 diabetes (**Figure 2B**). There was no difference in the level of hsa-miR-127-3p between the groups.

### Differentially Expressed Exosome-Enriched Extracellular Vesicle-Derived miRNAs Correlate Positively With HbA_1c_ in Mothers With Type 1 Diabetes

We next investigated the effect of the differentially expressed miRNAs on the clinical characteristics, BMI, blood glucose and HbA_1c_. Of the 176 differentially expressed miRNAs, 5 miRNAs showed a positive correlation to HbA_1c_ in mothers with type 1 diabetes (hsa-miR-6839-5p; hsa-miR-891a-5p; hsa-miR-1260a; hsa-miR-7977; hsa-miR-874-3p) after adjusting for multiple testing with Benjamini-Hochberg procedure, with a positive correlation above 0.6 (adj.p<0.05; **Supplementary Table 1**). However, as HbA_1c_ is known to be highly affected after delivery, introducing a bias to this observation, we did not pursue further analysis of these candidates. There was no significant association observed for miRNAs with BMI and blood glucose levels of the mothers with type 1 diabetes.

### Network and Pathway Analysis of miRNA Targets

**Figure 3:**
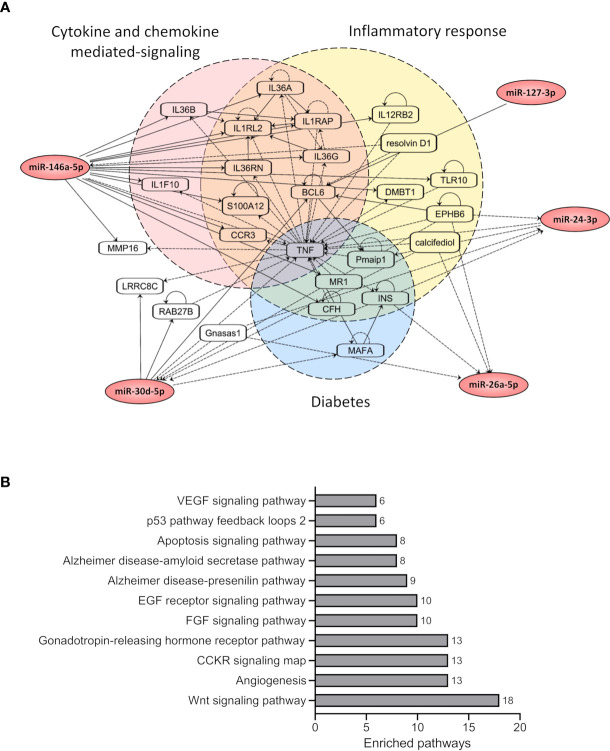
Network and pathway-based analysis of miRNA targets revealed enrichment for cellular signaling pathways. **(A)** The figure shows the network of the target genes associated with selected 5 miRNAs (hsa-miR-146a-5p, hsa-miR-26a-5p, hsa-miR-24-3p, hsa-miR-30d-5p, and hsa-miR-127-3p). The target genes were grouped based on their pathway annotations in IPA. **(B)** The figure shows enriched pathways associated with the target genes of the selected 5 miRNAs based on PantherDB annotations. Only significant pathways with FDR <0.05 are shown. The total number of target genes from the input list associated with each enriched pathway are also shown.

To understand the potential molecular interactions for the 5 selected miRNAs (hsa-miR-127-3p, hsa-miR-146a-5p, hsa-miR-26a-5p, hsa-miR-24-3p and hsa-miR-30d-5p), we performed a network-based analysis using IPA (**Figure 3A**). IPA network analysis identified a network consisting of 26 nodes (genes) and 79 edges (interactions). For the 5 miRNAs we used IPA Path Designer tool to find the interacting partners which were grouped into three main pathways, i) Inflammatory response, ii) Cytokine and Chemokine mediated signaling and iii) Diabetes, based on their functional annotations. The diabetes-related notes for the 5 miRNAs were: the major histocompatibility complex class I-related gene protein (MR1), MAF bZIP transcription factor A (MAFA), insulin (INS), complement factor H (CFH), phorbol-12-myristate-13-acetate-induced protein 1 (pmaip1) and tumor necrosis factor (TNF). The entire network had a core around TNF, with 35 edges associated to TNF alone.

We next performed pathway-based enrichment analysis of all target genes for the 5 selected miRNAs using PantherDB annotations. In total, 431 targets commonly identified by both TargetScan and miRTarBase were used for the pathway analysis (**Figure 3B**). The pathway analysis identified 11 significantly enriched pathways for the selected miRNA target genes. The top two significant pathways included Angiogenesis and CCKR signaling, further supporting the results from IPA analysis, suggesting a potential role of these miRNAs in cytokine-mediated inflammatory responses.

## Discussion

In the present study, we analyzed the miRNA profiles derived from plasma exosome-enriched extracellular vesicles by a newly developed sequence-based technique (QIAseq) designed to detect each copy of the miRNA present, with high specificity, sensitivity and dynamic range, through the integration of UMIs (35). QIAseq is one of the most efficient sequencing methods for miRNAs. It provides higher miRNA enrichment than other available small RNA sequencing technologies (36). With this method, we detected a total of 2,289 miRNAs and identified 176 differentially regulated miRNAs in plasma from lactating mothers with type 1 diabetes as compared to lactating healthy mothers around 5 weeks postpartum. Of these, 167 miRNAs were significantly upregulated and 9 were downregulated in the mothers with type 1 diabetes.

Five significantly upregulated candidates, known to be involved in diabetes and play roles in beta- and immune-cell functions: hsa-miR-127-3p, hsa-miR-146a-5p, hsa-miR-26a-5p, hsa-miR-24-3p and hsa-miR-30d-5p, were further investigated. By pathway analysis, we found these miRNAs to be associated with inflammatory response, chemokine and cytokine mediated signaling and diabetes-related pathways, with a core around TNF, a known modulator of immune response and a key player in immune-mediated diseases, including type 1 diabetes (37, 38).

Disease-associated circulating miRNAs have been suggested to carry direct clinical relevance, as they can be extracted from conventionally collected blood samples in the clinic, with the potential of improving disease prediction, diagnosis and stratification (39, 40). Thus, analyzing and interpreting the circulating miRNA signatures may constitute a feasible clinical application in the future. By real-time qPCR, we confirmed the significant upregulation of hsa-miR-146a-5p, hsa-miR-26a-5p, hsa-miR-24-3p and hsa-miR-30d-5p in plasma samples from mothers with type 1 diabetes, compared to healthy control mothers. The expression level of hsa-miR-127-3p, however, was unchanged in plasma between the two groups, even though this miRNA was significantly upregulated in the extracellular vesicles from mothers with type 1 diabetes. This discrepancy could be explained by the expression of distinct repertoires of some miRNA species in the respective biofluids of exosomes, which has previously been highlighted (41, 42). hsa-miR-127a-3p has also been found upregulated in the plasma extracellular vesicle fractions rather than free in plasma in individuals with Hodgkin lymphoma (42). Hence, the potential biomarker value of circulating hsa-miR-127-3p seems restricted to the extracellular vesicles. Previously, hsa-miR-127a-3p was found enriched in human pancreatic islets as compared to liver and skeletal muscle, where it was associated with insulin secretion and beta-cell function (43).

Consistent with the present pathway analysis, other studies highlight the role of miR-26a-5p in inflammatory response in several immune-mediated diseases (44–46), including type 1 diabetes pathogenesis (47). Circulating levels of hsa-miR-26a have previously been found upregulated in serum of children with newly diagnosed type 1 diabetes (18). Consistently, another study found it upregulated in plasma and showed a positive correlation of hsa-miR-26a-5p to HbA_1c_ at type 1 diabetes diagnosis (48).

Also miR-146a-5p has been well-studied for its role as an immune modulator in adaptive and innate immune responses, inflammation and apoptosis (16, 49–51), confirming the present study’s pathway analysis, i.e. hsa-miR-146a-5p’s association to inflammatory responses and chemokine and cytokine mediated signaling pathways. miR-146a-5p is expressed in various immune cells, participating in the regulation of inflammatory response, and has also been found to be upregulated by pro-inflammatory cytokines in human pancreatic islets (51, 52). Several studies found hsa-miR-146a-5p to be modulated in plasma and serum from individuals with type 1 diabetes (16, 53, 54). Polymorphisms in the gene encoding this miRNA have also been investigated for its protective role for type 1 diabetes, comparing the genotype of individuals with and without type 1 diabetes (55).

Altered levels of circulating hsa-miR-24-3p have been reported in newly diagnosed type 1 diabetes (54, 56). However, one study found hsa-miR-24-3p to be upregulated at newly diagnosed type 1 diabetes but found no difference at later stage type 1 diabetes, when compared to healthy controls (57). We have previously reported correlations between the plasma level of both hsa-miR-24-3p and hsa-miR-146a-5p to residual beta-cell function in children 6-12 months after diagnosis with type 1 diabetes (17). Furthermore, miR-24-3p has been associated with beta-cell failure, as overexpression of mmu-miR-24-3p in MIN6 cells inhibited beta-cell proliferation and insulin secretion (58).

Also, miR-30d has been shown to regulate insulin, as its overexpression in MIN6 cells induces insulin gene expression and silencing miR-30d inhibits glucose stimulated insulin expression (59). hsa-miR-30d has been studied in circulation, however, mostly in relation to type 2 diabetes, where it is upregulated in serum and plasma (54). Interestingly, one study found hsa-miR-30d-5p to be significantly upregulated in plasma from individuals with gestational diabetes (60).

In this study, we also observed that hsa-miR-6839-5p, hsa-miR-891a-5p, hsa-miR-1260a, hsa-miR-7977 and hsa-miR-874-3p positively correlated to HbA_1c_ in the group of mothers with type 1 diabetes. These have not previously been reported as markers for HbA_1c_. However, due to known effects on HbA_1c_ postdelivery, the biomarker potential of these miRNAs remains uncertain. Further analyses are needed to determine associations of these candidates to HbA_1c_.

Previously, we profiled miRNAs derived from exosome-enriched extracellular vesicles in breast milk from mothers with type 1 diabetes and healthy control mothers (13). Here, we found that miRNA levels in breast milk were not reflected in plasma. Only one candidate, hsa-miR-133a-3p exclusively overlapped between the two datasets, being significantly upregulated in both milk and plasma from the mothers with type 1 diabetes (13). However, while this differential expression was highly significant in milk, it was only nominally changed in the plasma samples. Overall, we conclude that the miRNA profiles depicted in these tissues hold distinct signatures, highlighting the biofluid or tissue-specific biomarker value of miRNAs (41).

From a general comparison, there are both discrepancies and similarities between our current findings and other studies investigating the miRNA profiles of individuals with type 1 diabetes. Garcia-Contreras et al. and Tesovnik et al. are to our knowledge the only studies that have investigated miRNAs verified to be derived from extracellular vesicles from plasma of individuals with type 1 diabetes (14, 15). The design and technologies used, however, differ greatly, giving rise to distinct identification profiles and quantities of detected miRNAs. These discrepancies most likely reflect the dynamic nature of miRNAs. Inconsistencies in design and methodologies are apparent in other studies investigating circulating miRNAs from individuals with and without type 1 diabetes (54, 61). Some candidates, however, are recurrent between studies and overlap with the present study’s findings, e.g. upregulation of hsa-miR-21-3p, hsa-miR-21-5p, hsa-miR-24-3p, hsa-miR-25-3p and hsa-miR-93-5p in plasma of individuals with type 1 diabetes compared to healthy controls (54, 61).

The design of the present study poses the risk that the phenotype under study could have considerable impact on immune state and disease status, reducing the chance to observe diabetes-related differences between the two study groups. Despite this limitation, we succeeded in detecting significant alterations in the miRNA profile between lactating mothers with and without type 1 diabetes, including miRNA candidates with positive correlations to HbA_1c_ and associations to disease pathogenesis and inflammatory responses. These observations should be verified in a supplementary cohort of lactating mothers with and without type 1 diabetes, ideally including continuous follow-up sampling before pregnancy, before and after delivery, and/or sampling from the infant. Hence, further such analyses are left to explain the full impact of altered circulating and exosome-entrapped miRNAs on lactating mothers with type 1 diabetes, as well as its potential of modulating type 1 diabetes risk in offspring. The results of this study further warrant investigations to ascribe functional importance of these miRNAs, and to elucidate the overall consequence of their alteration in type 1 diabetes.

## Data Availability Statement

The datasets presented in this study can be found in online repositories. The names of the repository/repositories and accession number(s) can be found below: https://www.ebi.ac.uk/arrayexpress/, E-MTAB-10458.

## Ethics Statement

The studies involving human participants were reviewed and approved by The Ethical Committee for the Capital Region, Denmark (H-4-2013-008). The patients/participants provided their written informed consent to participate in this study.

## Author Contributions 

SK and FP conceived and managed the study. LBN, HBM, and FP suggested the overall hypothesis and contributed to study design. LBN, ERM, PD, and JSv recruited participants and assembled phenotypic data. CF, AHM, RY, CE, JSt, JJ, FP, and SK carried out the sample preparation and interpretation of the data. HBM and FP secured resources and facilities for the research. CF, AHM, FP, and SK analyzed the data and drafted the manuscript. All authors provided critical scientific input and revised the manuscript. FP and SK had full access to all the data in the study and takes full responsibility for the integrity of the data and the accuracy of the data analysis. All authors contributed to the article and approved the submitted version.

## Funding

The study was supported by grants from the Region H (to HBM), the Novo Nordisk Foundation (to FP), The Beckett Foundation (to LBN), Aase og Ejnar Danielsens Fond (to AHM), The Sehested Hansen Foundation (to CF), Dronning Louises Børnehospitals Forskningsfond (to CF) and Frimodt-Heineke Fonden (to CF). Part of the research is supported by IMI2-EU under grant agreement No 115797 (INNODIA) and No 945268 (INNODIA HARVEST) (to FP). This Joint Undertaking receives support from the Union’s Horizon 2020 research and innovation program and “EFPIA”, “JDRF” and “The Leona M. and Harry B. Helmsley Charitable Trust”.

## Conflict of Interest

The authors declare that the research was conducted in the absence of any commercial or financial relationships that could be construed as a potential conflict of interest.

## Publisher’s Note

All claims expressed in this article are solely those of the authors and do not necessarily represent those of their affiliated organizations, or those of the publisher, the editors and the reviewers. Any product that may be evaluated in this article, or claim that may be made by its manufacturer, is not guaranteed or endorsed by the publisher.

## Abbreviations

ARF6, ADP-ribosylation factor 6; CFH, complement factor H; HSP70, heat shock protein 70; INS, insulin; IPA, Ingenuity Pathway Analysis; MAFA, MAF bZIP transcription factor A; MDS, multidimensional scaling; miRNA, microRNA; MR1, major histocompatibility complex class I-related gene protein; NTA, nanoparticle tracking analysis; Pmaip1, phorbol-12-myristate-13-acetate-induced protein 1; RNA-Seq, RNA-sequencing; TEM, transmission electron microscopy; TNF, tumor necrosis factor; UMIs, unique molecular identifiers.
