# Supplementary material for: Plasma Exosome-Enriched Extracellular Vesicles From Lactating Mothers With Type 1 Diabetes Contain Aberrant Levels of miRNAs During the Postpartum Period
Source: Front Immunol. 2021 Oct 8;12:744509. doi: 10.3389/fimmu.2021.744509 (PMC8531745; doi:10.3389/fimmu.2021.744509)
Supplement: Supplementary file 1 [file DataSheet_1.pdf]

Supplementary Material

Supplementary Tables

Supplementary Table 1: Pearson's correlation test of miRNAs and HbA_{1c} in the mothers with type 1 diabetes (n=26), two-tailed correlation(r); $p<0.05$ and with correction for multiple testing using Benjamini-Hochberg procedure (adj. p-value).

**miRNA correlation to HbA_{1c} in mothers with type 1 diabetes**			
**miRNA name**	**Correlation (r)**	**p-value**	**Adj p-value**
hsa-miR-6839-5p	0.710	0.00005	0.0086
hsa-miR-891a-5p	0.657	0.00027	0.0238
hsa-miR-1260a	0.656	0.00027	0.0161
hsa-miR-7977	0.637	0.00047	0.0208
hsa-miR-874-3p	0.633	0.00052	0.0183

Supplementary Figures

A

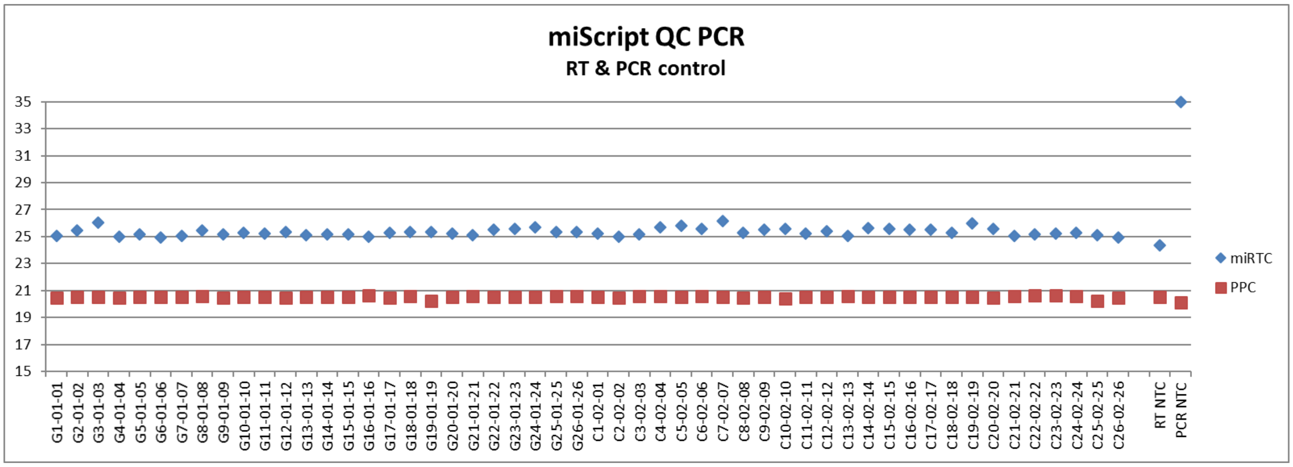

B

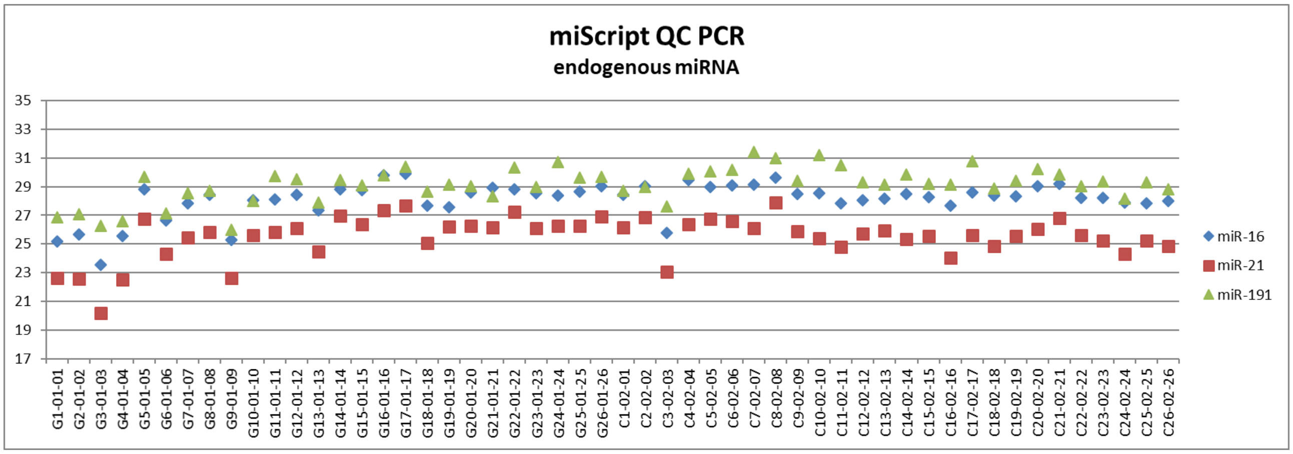

Supplementary Figure 1. The assessment of RNA extracted from exosome-enriched extracellular vesicles. (A) Quality control (QC) of RNA extracted from exosome-enriched extracellular vesicles by assessment of reverse transcription (RT) and PCR performances using miRTC miScript Primer Assay and positive PCR control (PPC) assay. (B) The RNA sample quality was further assessed by measured expression of ubiquitously expressed miR-16, miR-21, and miR-191 using miScript miRNA QC PCR Array. These miRNAs are known to be ubiquitously expressed in a wide range of cells, tissues and body fluid samples (including whole blood, serum, and plasma).

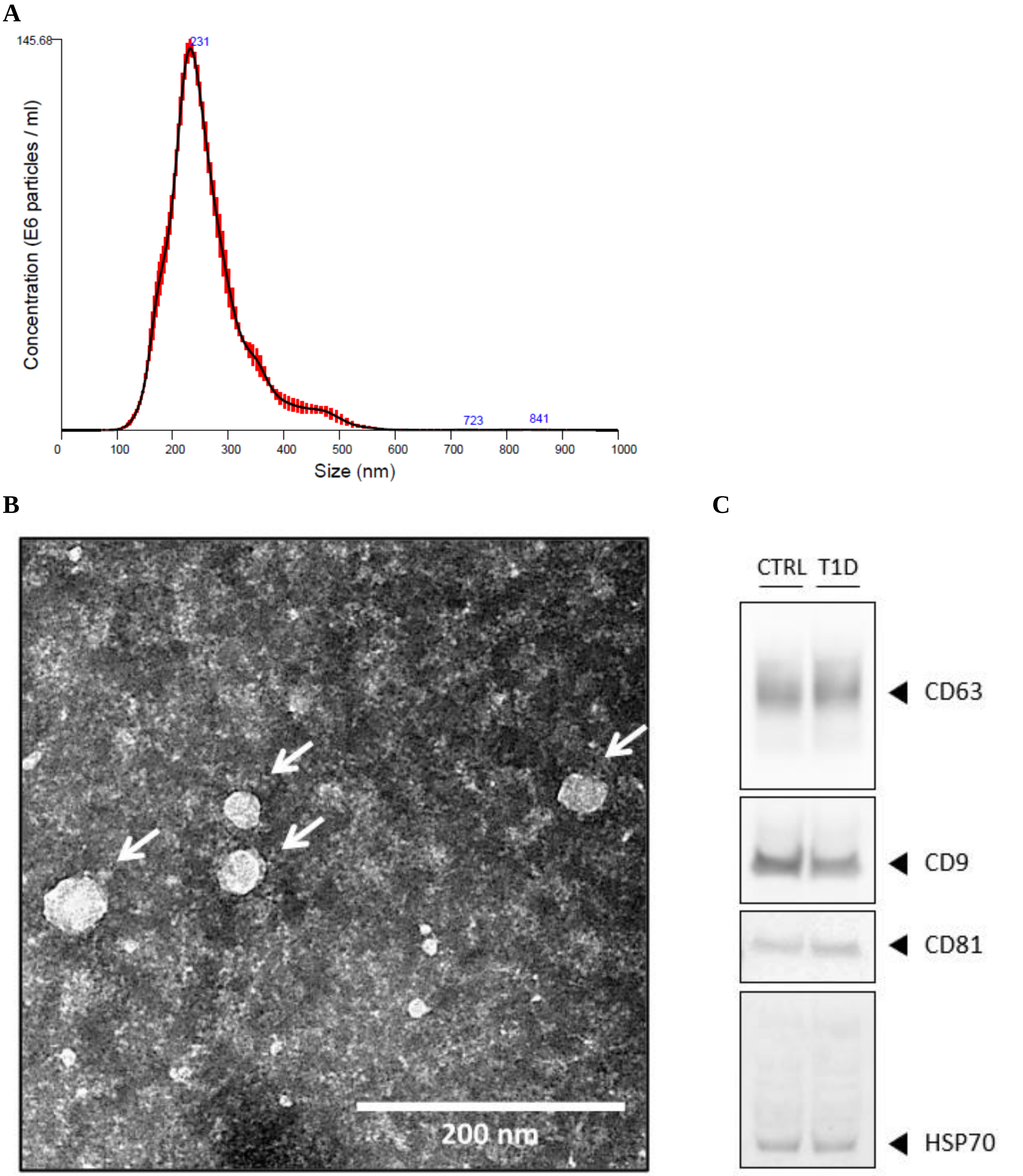

Supplementary Figure 2. Characterization of exosome-enriched extracellular vesicles derived from plasma samples. (A) Particle concentration per ml and size distribution (nm) +/- 1 standard error of the mean (red) analyzed by Nanosight NTA analysis. (B) Transmission electron microscopy of freshly prepared plasma-derived extracellular vesicles (indicated by arrows) by staining with 2% phosphotungstic acid. (C) Western blots of CD63 (30-60 kDa), CD9 (25 kDa), CD81 (26 kDa) and HSP70 (70 kDa) on plasma-derived extracellular vesicles from healthy control mothers (CTRL) or mothers with type 1 diabetes (T1D).

A

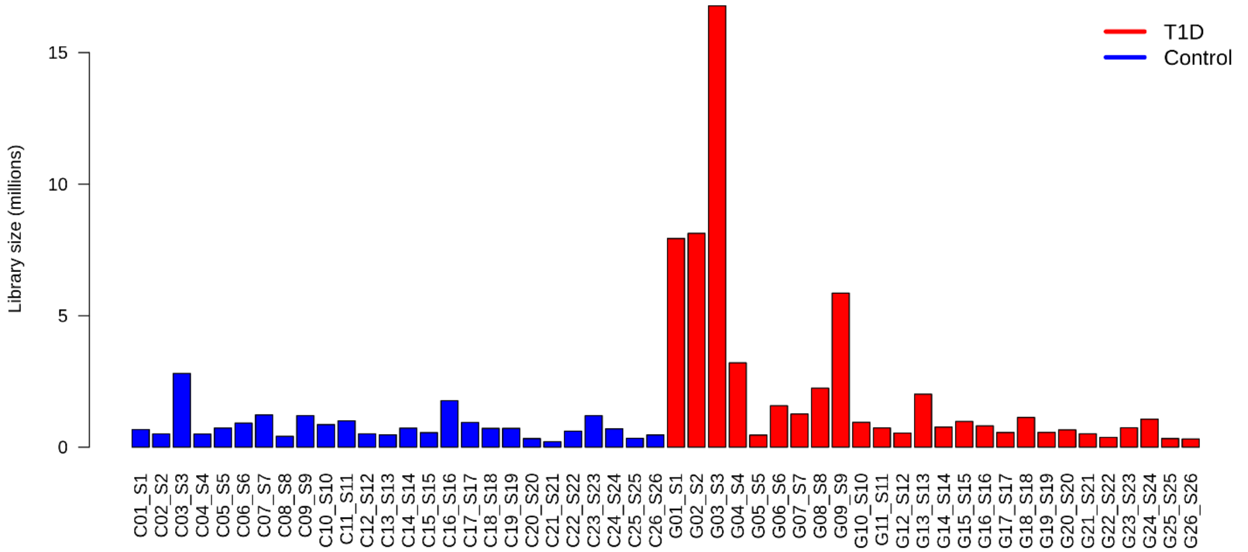
T1D
Control
Library size (millions)
0
5
10
15
C01_S1 C02_S2 C03_S3 C04_S4 C05_S5 C06_S6 C07_S7 C08_S8 C09_S9 C10_S10 C11_S11 C12_S12 C13_S13 C14_S14 C15_S15 C16_S16 C17_S17 C18_S18 C19_S19 C20_S20 C21_S21 C22_S22 C23_S23 C24_S24 C25_S25 C26_S26 G01_S1 G02_S2 G03_S3 G04_S4 G05_S5 G06_S6 G07_S7 G08_S8 G09_S9 G10_S10 G11_S11 G12_S12 G13_S13 G14_S14 G15_S15 G16_S16 G17_S17 G18_S18 G19_S19 G20_S20 G21_S21 G22_S22 G23_S23 G24_S24 G25_S25 G26_S26

B

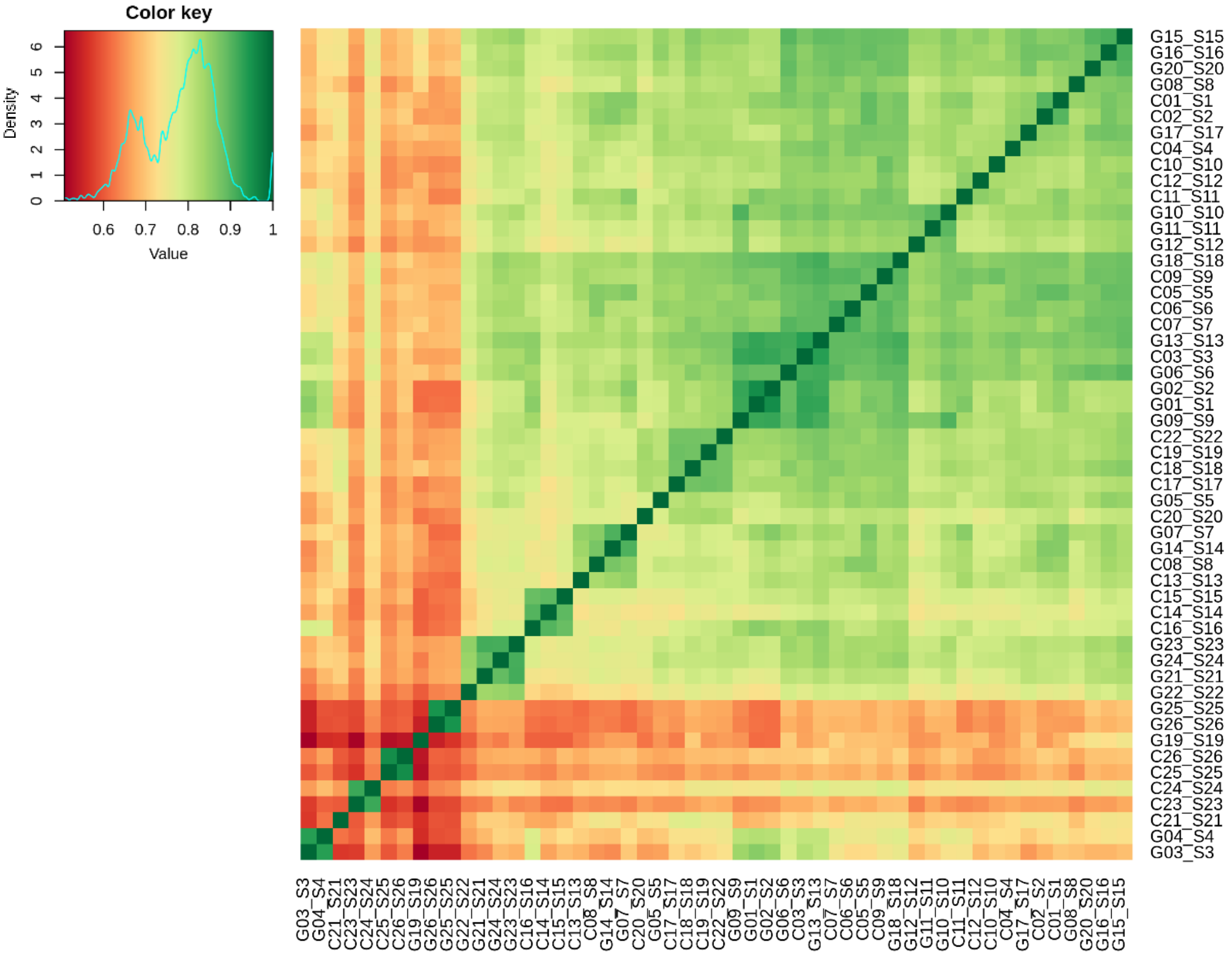
Color key
Density
Value
0.6
0.7
0.8
0.9
1
G15_S15 G16_S16 G20_S20 G08_S8 C01_S1 C02_S2 G17_S17 C04_S4 C10_S10 C12_S12 C11_S11 G10_S10 G11_S11 G12_S12 G18_S18 C09_S9 C05_S5 C06_S6 C07_S7 G13_S13 C03_S3 G06_S6 G02_S2 G01_S1 G09_S9 C22_S22 C19_S19 C18_S18 C17_S17 G05_S5 C20_S20 G07_S7 G14_S14 C08_S8 C13_S13 C15_S15 C14_S14 C16_S16 G23_S23 G24_S24 G21_S21 G22_S22 G25_S25 G26_S26 G19_S19 C26_S26 C25_S25 C24_S24 C23_S23 C21_S21 G04_S4 G03_S3

C

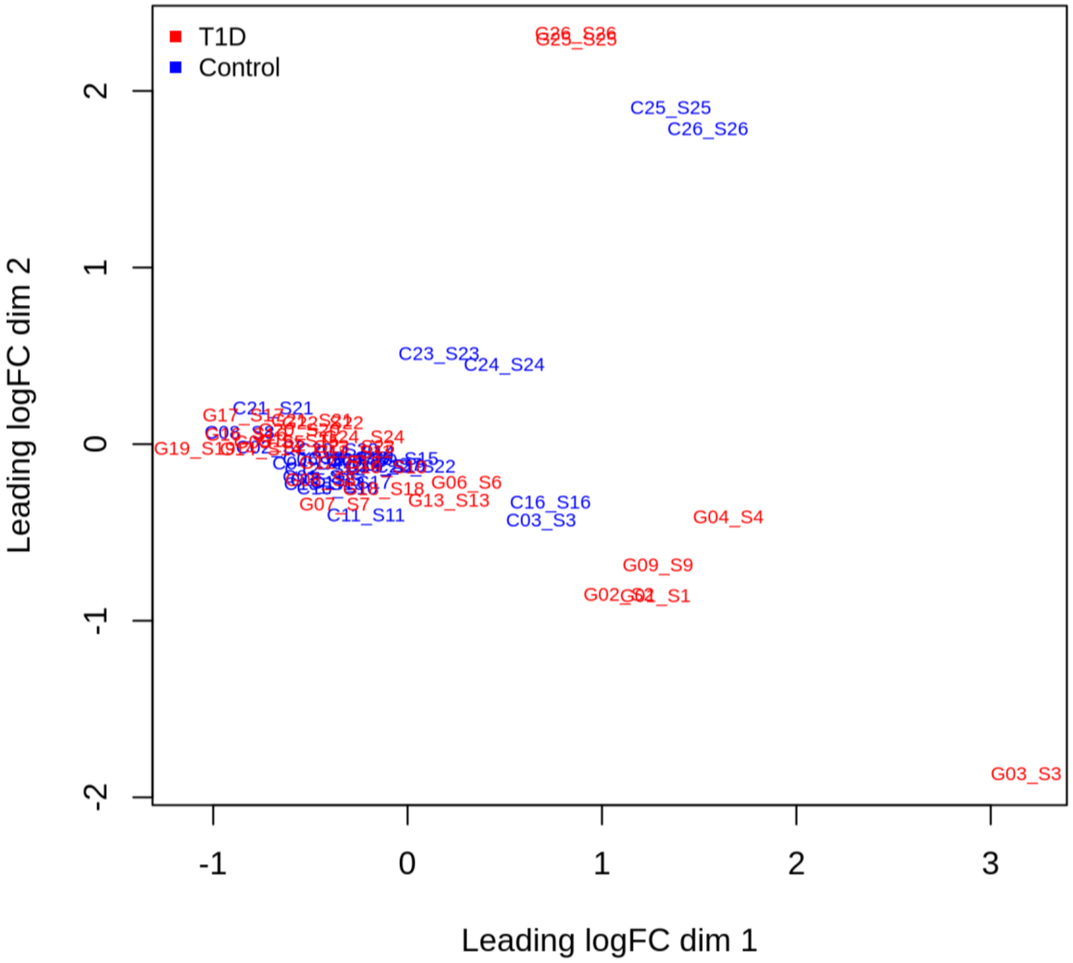

Supplementary Figure 3. Library size and sample correlations. (A) The bar plot shows the UMI reads distribution after small RNA sequencing for each sample. The type 1 diabetes (T1D) and control libraries are denoted by red and blue bars, respectively. (B) The correlation heatmap illustrates the relationship between each library for both T1D and control groups. Green indicates a close correlation while red indicates a more distant correlation in the data. (C) Multidimensional scaling (MDS) plot shows biological variability between the T1D and control group libraries.
